# Experimental and Constitutive Model on Dynamic Compressive Mechanical Properties of Entangled Metallic Wire Material under Low-Velocity Impact

**DOI:** 10.3390/ma13061396

**Published:** 2020-03-19

**Authors:** Yiwan Wu, Shangzhou Li, Hongbai Bai, Lei Jiang, Hu Cheng

**Affiliations:** Engineering Research Center for Metal Rubber, School of Mechanical Engineering and Automation, Fuzhou University, Fuzhou 350116, China; lishangzhoufzu@163.com (S.L.); bhbk11@sina.com (H.B.); jiangleixw@163.com (L.J.); chenghu2097@163.com (H.C.)

**Keywords:** entangled metallic wire material, porous material, low-velocity impact, mechanical properties, energy absorption capability, constitutive model

## Abstract

In this paper, the dynamic compressive mechanical properties of entangled metallic wire material (EMWM) under low-velocity impact were investigated and the constitutive model for EMWM under low-velocity impact was established. The research in this paper is based on a series of drop-hammer tests. The results show that the energy absorption rate of EMWM is in the range from 50% to 85%. Moreover, the EMWM with a higher relative density would not plastically deform macroscopically and has excellent characteristics of repetitive energy absorption. With the increase in relative density, the maximum deformation of EMWM decreases gradually, and the impact force of EMWM increases gradually. With the increase in impact-velocity, the phenomenon of stiffness softening before reaching the maximum deformation of EMWM becomes more significant. A constitutive model for EMWM based on the Sherwood–Frost model was established to predict the dynamic compressive mechanical properties of EMWM. The accuracy of the model was verified by comparing the calculated results with the experimental data of the EMWM with different relative densities under different impact-velocities. The comparison results show that the established model can properly predict the dynamic compressive mechanical characteristics of EMWM under low-velocity impact loading.

## 1. Introduction

Entangled metallic wire material (EMWM) is a novel porous material made of wire through a series of special processes. It can dissipate vibration energy through dry friction between adjacent wire helixes [1,2]. Some researchers also use the term metal rubber (MR) [3], metal wire mesh (MWM) [4], or elastic porous wire mesh (EPWM) [5]. However, they are the same kind of material because the manufacturing process and mechanical properties of these materials are highly similar. Compared to traditional polymer material (such as natural rubber), the EMWM has some outstanding advantages, such as high-low temperature resistance and corrosion resistance. Therefore, EMWM has been widely used in extreme environments, such as the vibration reduction in ship’s foundations under high temperature [6], the micro-vibration isolation system of spaceborne cryocoolers [7], and the sealing of rotors for turbo-machinery [8].

Porous materials, such as metal foams and honeycomb materials, can effectively absorb impact energy and have attracted the attention of many researchers and material manufacturers. These porous materials are “hard frame materials” (HFMs). The edge of the cell element of a HFM is a rigid beam or face, and the connection between the cell elements is a rigid connection. When a HFM is subjected to impact load, the impact energy will be absorbed through plastic deformation of the edges of the cell elements of the HFM. Therefore, the HFM cannot dissipate impact energy repeatedly because of irreversible plastic deformation. The EMWM is a kind of open-cell “soft frame material” (SFM) [9]. The edge of the cell element of EMWM is variable edge, which is determined by the contact point between adjacent wire helixes. When EMWM is subjected to impact load, the impact energy will be absorbed through dry friction between the adjacent wire helixes and air damping caused by air discharge and inhalation of internal pores [9]. Wu et al. [9] noted that the EMWM can return to its original size under multiple impact loads. However, when the impact load exceeds the bearing limit of the EMWM, the EMWM may be molded again. This means that the EMWM will plastically deform.

In previous research, the researchers’ investigations on EMWM mainly focused on the quasi-static mechanical properties [10,11,12], dynamic mechanical properties [13,14,15], the influence of different factors on its mechanical properties [16,17,18,19,20,21], damping mechanism [1,22] and engineering application [23,24]. However, the dynamic compressive mechanical properties of entangled metallic wire material under low-velocity impact have not received enough attention from researchers. Liu et al. [25] studied a kind of sintered entangled metallic wire material (SEMWM) under impact load and found that the impact toughness of SEMWM increases with the decrease in its porosity. The internal adjacent wires of the SEMWM were sintered together at the point of contact through a vacuum furnace. Therefore, the SEMWM is a kind of “hard frame material”. Guérard et al. [26] investigated the mechanical properties of a single wire entangled material with different high strain rate (by a Hopkinson bar device). The results of their experiments show that the strain rate and density have a significant influence on the dynamic mechanical properties of EMWM. The results of Guérard et al. also confirm that the EMWM has good suitability for impact energy absorption but did not analyze the impact energy dissipation mechanism of EMWM. Xia et al. [27] carried out theoretical and experimental research on the shock protection characteristics of two types of EMWM isolator. Liu et al. [28] applied the EMWM to the gun’s latch block buffer, and their test results show that the cushion device made by EMWM has better properties and life on impact environment than that made by polymer materials. Jeong et al. [29] designed a frequency tunable vibration and shock isolator with mesh washer, and the results from their experimental reveal that the isolator can not only achieve the performance of shock attenuation and but also can avoid the vibration amplification. Recently, Wu et al. [9] investigated the mechanical behavior of EMWM under quasi-static and low-velocity impact loading and noted that the EMWM has excellent characteristics of repetitive energy absorption. However, the influence of material parameters on its energy absorption performance was not considered by Wu et al. [9]

To facilitate the application of EMWM for shock absorption, it is necessary to investigate the constitutive model of EMWM, which can predict the dynamic compressive mechanical properties of EMWM under impact loading. Due to the complex internal spatial structure of EMWM, it is difficult to establish a constitutive model of EMWM with the consideration of the actual internal space structure. Therefore, many scholars have adopted statistical analysis and parameter fitting methods to obtain macro-mechanical models of EMWM based on experiments [30,31]. The Sherwood-Frost model [32], as shown in Equation (1), was proposed by Sherwood and Frost in the 1990s, and has often been used to establish the constitutive models of various metal foams [33,34,35,36]. By comparing and analyzing the constitutive model of metal foam and EMWM, Li et al. [37] presented a constitutive model for knitted-dapped EMWM by using the Sherwood–Frost model and performed parameter fitting. Ding et al. [38] proposed a modified constitutive model for plate-like EMWM with the consideration of thermal expansion.

(1) $$\sigma_{eq}=H(T)G(\rho )M(\varepsilon ,\dot{\varepsilon })f(\varepsilon )$$

where σ*_eq_* is the equivalent stress, *H*(*T*) is the temperature softening term, *G*(*ρ*) is the density term, *ρ* is the density, *M*(*ε,*$\dot{\varepsilon }$) is the strain rate enhancement term, and *f*(*ε*) is the shape function.

The EMWM is a kind of porous material with energy absorption and excellent impact resistance. The dynamic compressive mechanical properties of EMWM under low-velocity impact will be investigated by a series of drop-hammer tests. The effect of impact velocity and relative density on mechanical properties and energy absorption mechanism of EMWM will be studied. Finally, a constitutive model for EMWM will be proposed to predict the dynamic compressive mechanical properties of EMWM under low-velocity impact loading.

## 2. Experimental Methodology 

### 2.1. Materials Used and Specimen Preparation

In this paper, austenitic stainless-steel wires 304 (0Cr18Ni9) with a diameter of 0.3 mm were used as the raw material for manufacturing EMWM specimens. A three-step process was adopted to fabricate the EMWM specimens [1,9,10,26]. First, the straight austenitic stainless steel wire was processed into a dense wire helix according to the principle of coil spring processing; second, a rough porous base material of EMWM was prepared by fixed-pitch stretching and cross weaving of the tight wire helix; third, the base material was put into a specific mold, and molded to obtain an EMWM specimen.

Relative density is the most important structural parameter of porous materials [39,40], and is often used to evaluate the porosity in a porous material. The relative density of EMWM (*ρ_r_*) can be calculated as

(2) $$\rho_{r}=\frac{\rho }{\rho_{s}}=1-\varphi$$

where *ρ* is the density of EMWM, *ρ_s_* is the density of the base material, *ρ_s_* = 7.87 g/cm^3^, and *φ* is the porosity of EMWM.

**Figure 1:**
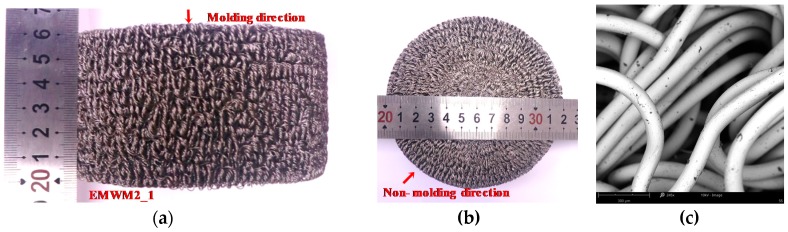
Entangled metallic wire material (EMWM) specimen’s views in (**a**) molding direction, (**b**) non-molding direction and (**c**) scanning electron microscope (SEM) image of EMWM (245× enlargement).

To investigate the influence of relative density on the dynamic performance of EMWM and assess the repeatability of the results, 4 batches of EMWM with different relative densities were manufactured, with each batch being composed of 5 specimens. One of the manufactured EMWM specimens is shown in Figure 1. The specific size parameters of the EMWM specimens are listed in Table 1, and CI is the confidence intervals of parameter values in 0.95 of the confidence.

### 2.2. Drop-Weight Impact Tests

**Figure 2:**
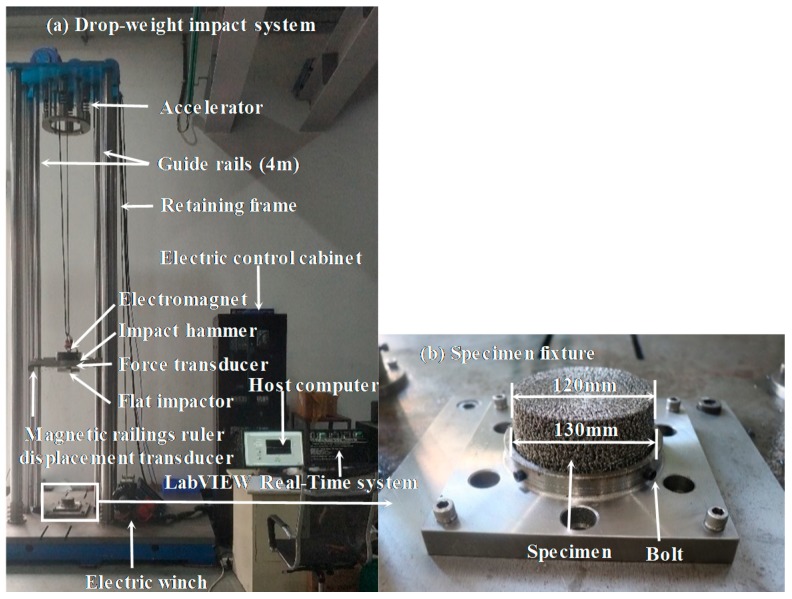
Experimental setup of low-velocity impact tests: (**a**) drop-weight impact system and (**b**) specimen fixture.

The dynamic compressive mechanical properties of EMWM under low-velocity impact was tested by a series of drop hammer tests. A self-designed drop hammer test device, which is shown in Figure 2a, was used to carry out the tests. The drop hammer test device was mainly composed of a hammer, a dynamic force sensor (YX-60T, Yi Xuan Electronic Technology Co., Ltd., Yangzhou, China), a displacement sensor (MTS-H10C, GIVI, Nova Milanese, Italy) and a real-time data acquisition and control system. The weight of the hammer was 76 kg. The contact surface between the drop hammer and the specimen was flat. The maximum lifting height of the drop hammer was 4 m. The real-time data acquisition and control system was built up on the bias of the LabVIEW-RT system and an X series data acquisition device (NI PCIe-6351, National Instruments, Austin, TX, USA). The maximum detection range of the YX-60T is from 0 to 600 kN. The displacement resolution of MTS-H10C is 10 μm. For this device, the force and magnetic sensors with different measuring range and accuracy can be replaced according to the testing needs. During the impact test, each EMWM specimen was installed in a pre-designed fixture, as shown in Figure 2b. The impact velocity and corresponding impact energy and initial strain rate are summarized in Table 2. The tests were divided into two parts. The first part entailed each sample being tested repeatedly from the lowest to the highest impact velocity, and the change in the sample height was recorded at the end of each test. In the second part, for the specimens whose height had changed after the first part of the test, the samples with the same parameters were reprepared and only a single impact test was carried out to compare the effects of different impact times on the mechanical properties and energy absorption properties of the specimens. 

To investigate the dynamic compressive mechanical properties and energy absorption of EMWM, specific energy absorption (*SEA*), energy absorption rate (*η_D_*) and impact stiffness (*k*) were derived from the experiment data.

According to the law of conservation of energy, total impact energy (*E_0_*) is defined as Equation (3).

(3) $$E_{0}=mgh=\frac{1}{2}mV_{0}^{2}$$

where *m* is the mass of the hammer, *m* = 76 kg; *g* is the acceleration of gravity, *g* = 9.8 m/s^2^; *h* is the lifting height of the drop hammer; and *V_0_* is the initial impact velocity.

The energy absorbed (*E_a_*) by EMWM can be expressed as follows:

(4) $$E_{a}=\frac{1}{2}mV_{0}^{2}-\frac{1}{2}mV_{1}^{2}$$

where *V*_1_ is the velocity of the drop-hammer at which the force is almost zero at the end of the unloading process.

The specific energy absorption (*SEA*) is the energy dissipated by the EMWM of per unit mass and is defined as Equation (5).

(5) $$SEA=\frac{E_{a}}{m_{r}}$$

where *m_r_* is the mass of EMWM specimen.

The energy absorption rate (*η_D_*) is used to evaluate the energy absorption capability of EMWM under low-velocity impact and can be calculated as

(6) $$\eta_{D}=\frac{E_{a}}{E_{0}}$$

The energy absorption rate (*η_D_*) can be obtained by the combination of Equations (3), (4) and (6). *η_D_* can be expressed as

(7) $$\eta_{D}=\frac{{V_{0}}^{2}-{V_{1}}^{2}}{{V_{0}}^{2}}$$

The impact stiffness (*k*) represents the loading-bearing capacity of EMWM. Its expression is as Equation (8)

(8) $$k=\frac{F_{\max}}{X_{\max}}$$

where *F*_max_ and *X*_max_ are the maximum force and maximum displacement, respectively.

To facilitate the establishment of the constitutive model for EMWM under low-velocity impact, the force-displacement curves of EMWM can be transformed into stress-strain curves. The stress (*σ*), strain (*ε*) and initial strain rate ($\dot{\varepsilon }$) can be calculated, respectively, as

(9) $$\sigma =\frac{F}{S}$$

(10) $$\varepsilon =\frac{x}{H}$$

(11) $$\dot{\varepsilon }=\frac{V_{0}}{H}$$

where *F* is the force, *S* is the cross-sectional area of EMWM specimen, *H* is the height of EMWM specimen, and *x* is the displacement.

## 3. Results and Discussion

### 3.1. Impact Process Analysis

**Figure 3:**
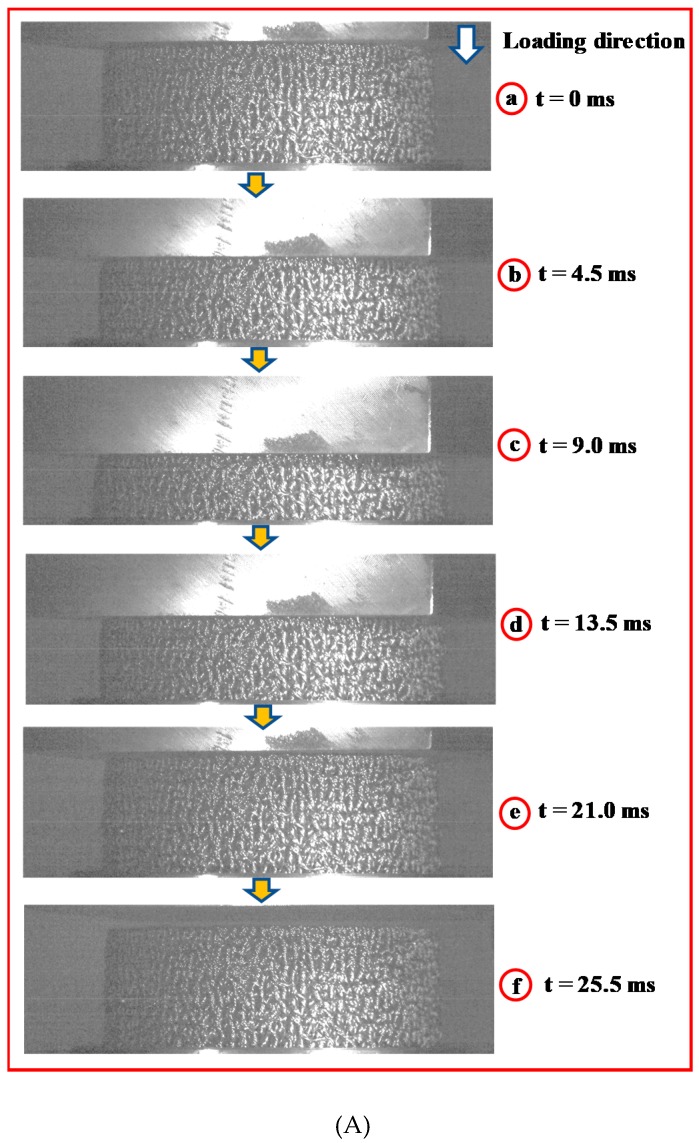

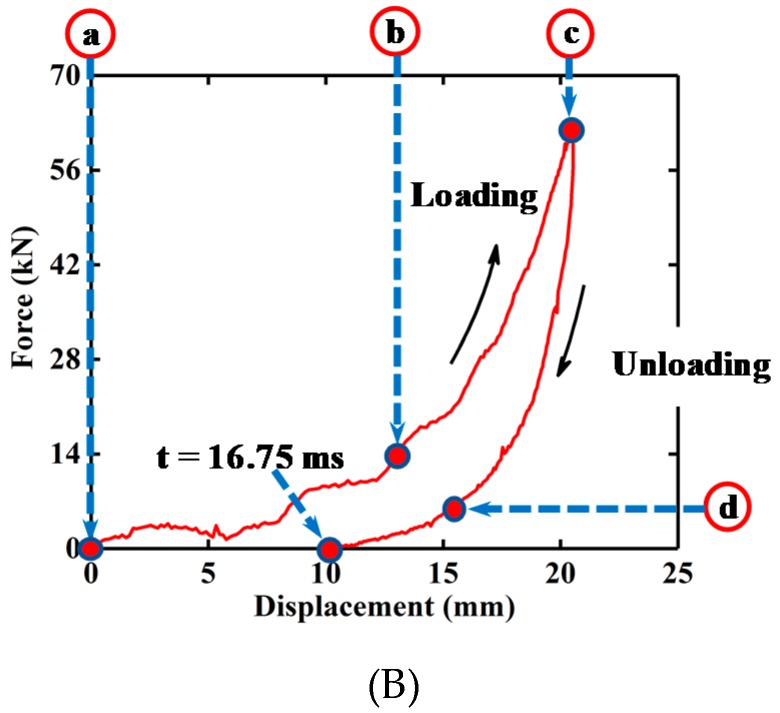
Dynamic compression processes of EMWM (*ρ_r_* = 0.25, *V*_0_ = 3 m/s): (**A**) Image of the compression processes and (**B**) Force-displacement of the compression processed.

A high-speed 10-bit CMOS camera (PCO.1200hs, PCO AG, Kelheim, Germany) was used to observe the entire impact process at a frame rate of 2000 f/s. The impact process of EMWM is shown in Figure 3. Figure 3a shows the moment when the drop hammer comes into contact with the EMWM specimen. At this moment (t = 0 ms), the kinetic energy of the drop hammer reaches the maximum. Figure 3b (t = 4.5 ms) shows the deformation of the EMWM specimen in the process of drop hammer compression. Figure 3c (t = 9.0 ms) shows the moment when the deformation of the EMWM specimen reaches the maximum. Figure 3d (t = 13.5 ms) shows the deformation of the EMWM in the process of recovery. Figure 3e shows the moment when the drop hammer and the EMWM specimen begin to separate. Figure 3f shows the moment when the EMWM specimen returns to its original position. Figure 3g presents the force–displacement curves of the homologous impact process. It can be seen from the impact process that the EMWM compresses and then recovers rapidly to its original position under low-velocity impact loading. In general, the EMWM can withstand repeated impacts at finite deformation or loading. A similar deformation mode of EMWM under quasi-static compression was also observed by Rodney et al. [41].

During the 0–9.0 ms period, the EMWM is compressed by the impact load and is significantly deformed. During this process, part of the impact energy is dissipated by the EMWM in the form of dry friction and air damping, and the rest is stored in the EMWM in the form of elastic potential energy. In addition, for low density EMWM, the plastic deformation of the wire helixes will also consume part of the impact energy. The EMWM is a kind porous material, and its inner pores are filled with air. When the EMWM is compressed, its internal air will be squeezed out. According to the theory of fluid mechanics, air resistance is proportional to the square of the relative velocity between the internal air and wire helixes. This means that the more severe the deformation of the EMWM is, the faster the internal air is extruded, and the greater the air damping generated by the EMWM. During the 9.0–21.0 ms period, the impact energy, which is stored in the form of elastic potential energy, is released. During this process, part of the elastic potential energy is dissipated by the EMWM in the form of dry friction and air damping, and the rest is converted into the kinetic energy of the drop-hammer. Similar to the principle of air damping generated by extruded air, there is also a damping effect when external air enters the internal pores of the EMWM. During the 21.0–25.5 ms period, the EMWM continues to recover its shape. During this process, residual elastic potential energy is continuously absorbed by the EMWM.

### 3.2. Force–Displacement Response

**Figure 4:**
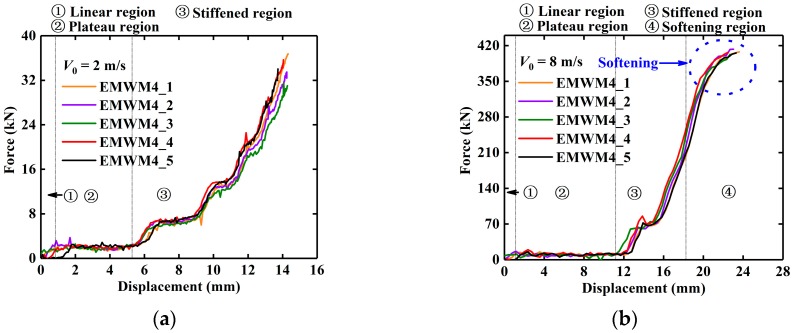
Force–displacement curve of EMWM during impact loading process: (**a**) without softening region and (**b**) with softening region.

Figure 4 presents two force-displacement curves of an EMWM specimen under different initial impact velocities. It can be seen from Figure 4a that the shape of the curve of the EMWM with a low impact velocity (2 m/s) is similar to that under quasi-static loading, and can be divided into three regions: linear region, plateau region and stiffened region [10]. As shown in Figure 4a, the slope of the force–displacement curve increases with the increase in deformation. However, the shape of the curve of the EMWM with a relatively high impact velocity (8 m/s) can be divided into four regions: linear region, plateau region, stiffened region and softening region. Stiffness softening occurs before maximum deformation is reached (blue dotted circle in Figure 4b). The reason for the difference in the shape of the curves is that the air damping is not obvious at a relatively low impact velocity. In the case of a relative-high velocity impact loading, the impact energy is dissipated by the EMWM in the form of plastic deformation, dry friction and air damping, and the deformation velocity of the EMWM will become slower until it reaches maximum deformation. This means that air damping will gradually decrease until it is zero.

**Figure 5:**
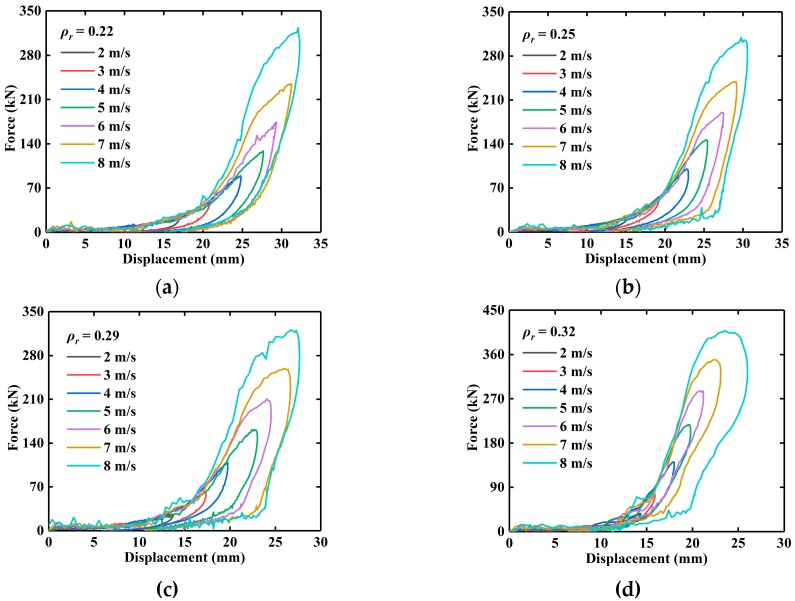
Experimental force-displacement curves of the EMWM with different relative densities under different impact velocities: (**a**) *ρ_r_* = 0.22, (**b**) *ρ_r_* =0.25, (**c**) *ρ_r_* = 0.29, and (**d**) *ρ_r_* = 0.32.

For each batch of EMWM, the test results of the EMWM specimens with the same relative density are similar. Therefore, only the force-displacement curve of one specimen of each batch was presented. Figure 5 shows the force–displacement hysteresis loops of the EMWM with different relative densities under different initial impact velocities. It is noted that the stiffness softening phenomenon is more obvious with the decrease in the relative density of EMWM. It can also be seen from Figure 5 that the maximum deformation of EMWM mainly depends on impact velocity and relative density. As impact velocity increases, the maximum deformation significantly increases.

**Figure 6:**
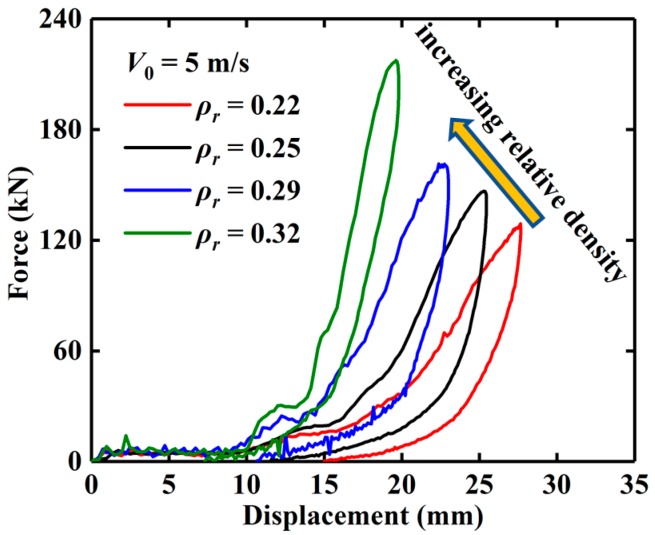
Experimental force–displacement curves of EMWM specimens under different relative densities.

Figure 6 presents the force–displacement curves of EMWM with different relative densities under 5 m/s impact. The maximum deformation of EMWM with different relative density is different under the same impact energy. It is noted that with the increase in relative density, the maximum deformation of EMWM decreases gradually, and the impact force of EMWM increases gradually. The reason for this is that the internal porosity of the EMWM with higher relative density is smaller, and the wire helix is more likely to extrude each other, so the maximum deformation will be reduced. On the other hand, at the same initial impact velocity (5 m/s), a smaller amount of deformation of EMWM means that the drop hammer bears a greater deceleration. Therefore, according to Newton’s second law, the maximum impact force of EMWM with small deformation is greater.

**Figure 7:**
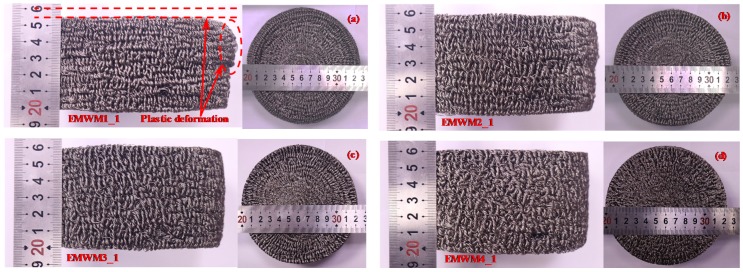
EMWM specimens after all experiment, specimen code: (**a**) EMWM1_1, (**b**) EMWM2_1, (**c**) EMWM3_1 and (**d**) EMWM4_1.

It is known from the forming process of EMWM that the EMWM is cold-formed under a specific external load. The forming process of EMWM is the plastic deformation process of metal wire helix. To fabricate a denser EMWM, a greater forming force must be applied. When the critical load is exceeded, the wire helix of the EMWM may be partially plastically deformed. Therefore, the EMWM with lower relative density is more prone to plastic deformation, as demonstrated in Figure 7. It can be seen that the extent of plastic deformation of EMWM decreases with the increase in relative density. The macroscopic plastic deformation of EMWM shows as follows: its molding direction height reduced, and non-molding direction expanded outward.

**Figure 8:**
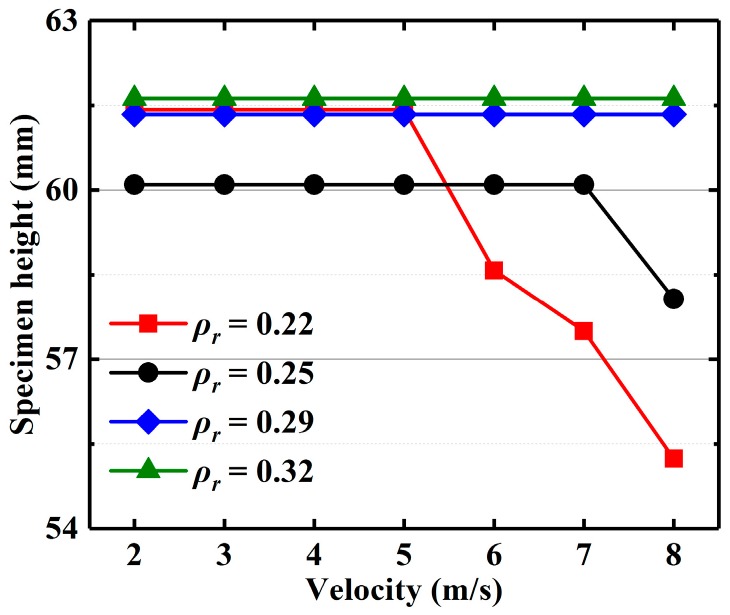
The height of EMWM specimen with different relative densities under different impact velocities.

Figure 8 presents the height variation curves of EMWM with different relative densities under different impact loadings. The EMWM with relative densities of 0.29 and 0.32 have no change in height at impact speeds from 2 to 8 m/s. This means that the two batches of EMWM specimens were not plastically deformed, and the impact energy is not dissipated through plastic deformation of the material, or is negligible. Meanwhile, the height of the EMWM with relative densities of 0.22 and 0.25 decreases with decreasing density at a relatively high impact velocity.

**Figure 9:**
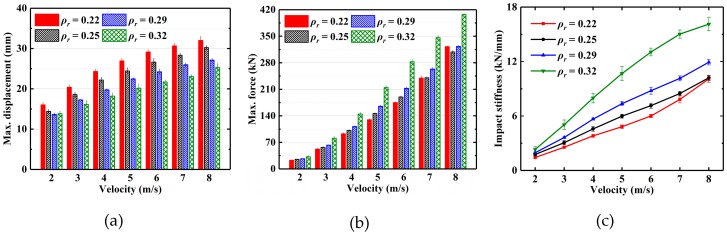
Comparison of experimentally measured (**a**) maximum displacement, (**b**) maximum force and (**c**) impact stiffness under different impact velocities.

The mean values and standard deviation of the maximum displacement and maximum force under different impact velocities are presented in Figure 9a,b. Figure 9c shows the mean values and standard deviation of impact stiffness under different impact velocities. As shown in Figure 9c, the impact stiffness increases with the increase in impact velocity, and the impact stiffness is linearly related to the impact velocity. Meanwhile, the impact stiffness of the EMWM with the relative density of 0.22 at 8 m/s increases significantly. This is caused by the plastic deformation of the EMWM with a lower relative density. At 8 m/s, the increasing trend of the impact stiffness of EMWM with the relative density of 0.32 slows down obviously.

### 3.3. Energy Absorption Characteristics

**Figure 10:**
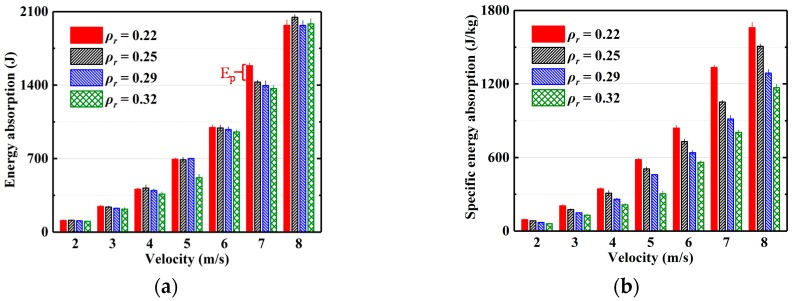
Comparison of experimentally measured (**a**) absolute energy absorption and (**b**) specific energy absorption under different impact velocities.

Based on the measured force–displacement curves, the energy absorbed (*E_a_*) by EMWM in the tests can be calculated according to Equation (4). The mean values and standard deviation of the absorbed energy and the corresponding specific energy absorption (*SEA*) of EMWM under different impact velocities are presented in Figure 10. It can be seen from Figure 10a that the impact energy absorbed by the EMWM (*ρ_r_* = 0.22, 0.25 and 0.29) is almost the same in the low-velocity impact tests (2, 3, 4, 5 and 6 m/s), while that absorbed by the EMWM (*ρ_r_* = 0.32) is the least. In the low-velocity impact tests (7 m/s), the energy absorption of EMWM (*ρ_r_* = 0.22) is significantly higher. However, in the low-velocity impact tests (8 m/s), the energy absorbed by the EMWM (*ρ_r_* = 0.22) is significantly reduced. The reason for this is that the EMWM (*ρ_r_* = 0.22) cannot maintain its original energy absorption ability after producing plastic deformation in the molding direction (6 and 7 m/s). Therefore, the EMWM may affect energy absorption after the plastic deformation. The red brace (E_p_) in Figure 10a represents the dissipated energy by the plastic deformation of EMWM. It can also be seen from Figure 10b that the specific energy absorption significantly decreases with the increase in relative density.

To investigate the effect of plastic deformation of EMWM on the mechanical properties and energy absorption, a new-batch EMWM specimen (*ρ_r_* = 0.22, 0.25) was manufactured to conduct a low-velocity impact test (7 m/s and 8 m/s). The results show that the energy absorption of EMWM subjected to a single impact is almost equal to that of EMWM subjected to accumulated impacts in the previous tests, which have undergone plastic deformation. The plastic deformations under accumulated impacts are summarized in Figure 8. The plastic deformation of EMWM with high density is negligible, so the effect of plastic deformation on its energy absorption properties can be ignored, and vice versa.

**Figure 11:**
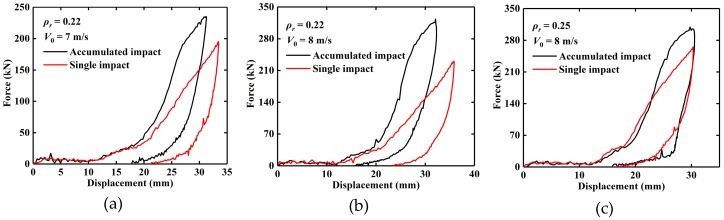
Experimental force–displacement curves of EMWM specimens after accumulated impact and single impact, (**a**) *ρ_r_* = 0.22, *V*_0_ = 7 m/s, (**b**) *ρ_r_* = 0.22, *V*_0_ = 8 m/s and (**c**) *ρ_r_* = 0.25, *V*_0_ = 8 m/s.

To analyze the influence of the plastic deformation of EMWM, the force-displacement curves under accumulated impact and single impact are shown in Figure 11. For EMWM with relatively low density, plastic deformation will occur after the accumulated impact, resulting in an increase in its stiffness. 

**Figure 12:**
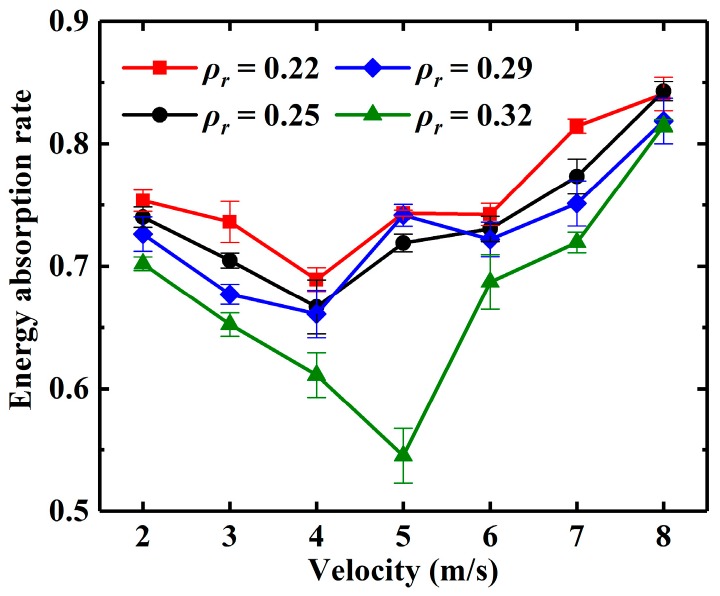
Energy absorption rate of EMWM specimens under different impact velocities.

The mean value (MV), standard deviation (STD) and confidence intervals (CI) in 0.95 of the confidence of the energy absorption rate of EMWM under different initial impact velocities are summarized in Table 3, Table 4, Table 5 and Table 6 and Figure 12. It can be observed that the energy absorption rates of EMWM with different relative densities are more than 0.5. The results show that the EMWM is a kind of material with high energy absorption rate. Especially when the relative density of EMWM is greater than a certain value, it will not undergo plastic deformation and can withstand repeated impacts.

As the impact velocity increases, the energy absorption rate first decreases and then increases at a critical point, which is shown in Figure 12. The critical point is 4 m/s when the relative density of EMWM is 0.22, 0.25 and 0.29. The critical point is 5 m/s when the relative density is 0.32. This phenomenon is caused by insufficient friction and air damping and has been explained in the author’s previous research [9].

It can be observed that the energy absorption rate of EMWM decreases with the increase in the relative density. There are two reasons to explain this phenomenon. First, as the relative density increases, the internal porosity of the EMWM becomes smaller, and the wire helices are more likely to extrude each other, which will result in a reduction in the amount of energy dissipated by friction. On the other hand, the increase in relative density will lead to a decrease in air content in EMWM and then the weakening of the air damping.

## 4. Constitutive Model

### 4.1. Modified Shape Function

Sherwood and Frost expressed the shape function *f*(*ε*) of polyurethane foam by power series. In their test, the maximum strain of polyurethane foam varies little under different strain rates, so it is appropriate to use power series to express the shape of the stress–strain curves. However, it is not appropriate to directly use power series as the shape function of EMWM under low-velocity impact. As shown in Figure 5, the maximum strain of EMWM varies greatly under different impact velocities.

Zheng et al. [42,43] proposed a dynamic material model with the dynamic plastic hardening function (D-R-PH), which was expressed as

(12) $$\sigma_{l}=\sigma_{0}^{d}+D\varepsilon /{\left(1-\varepsilon \right)}^{2}$$

where σ*_l_* is the effective principal stress, *D* is a fitting parameter and $\sigma_{0}^{d}$ is the dynamic initial crush stress. 

The advantage of the D-R-PH model is that the parameters are simple and have a high degree of consistency between the calculated result and experimental data. However, the D-R-PH model is rate-independent under dynamic compression process.

The shape function *f*(*ε*) = *ε*/(1 − *ε*)^2^ is a good description of the stress–strain trend for foam materials. After comparing with the test data from the dispersion degree and considering the particularity of EMWM, we find that the similar function *f*′(*ε*) is suitable for describing the stress–strain curves of EMWM under low-velocity impact. The modified shape function *f*′(*ε*) can be defined as

(13) $$f^{\prime }(\varepsilon )=\frac{D_{0}\varepsilon^{2}}{{\left(1-\varepsilon \right)}^{2}}$$

where *D*_0_ is an empirical fitting parameter. The value of *D*_0_ obtained by data fitting using the data of reference strain rate and reference relative density (${\dot{\varepsilon }}_{0}$ = 33.33 s^−1^, *ρ_r0_* = 0.22) is 13.95 MPa.

The constitutive model for EMWM under low-velocity impact can be initially obtained by the combination of Equations (1) and (13). It can be expressed as

(14) $$\sigma (\varepsilon )=H(T)M(\varepsilon ,\dot{\varepsilon })R(\rho_{r})f^{\prime }(\varepsilon )=M(\varepsilon ,\dot{\varepsilon })R(\rho_{r})\frac{D_{0}\varepsilon^{2}}{{\left(1-\varepsilon \right)}^{2}}$$

where *H*(*T*) is the temperature softening term, all tests were carried out at room temperature, *H(T)* = 1; *R*(*ρ_r_*) is the relative density term; *ρ_r_* is the relative density; $M(\varepsilon ,\dot{\varepsilon })$ is the strain rate enhancement term; and *f*′(*ε*) is the modified shape function.

### 4.2. Effect of the Relative Density

As mentioned above, the relative density has a significant influence on its mechanical behavior under low-velocity impact. The power function or linear function is often used to express the relationship between relative density and stress [32,37,38]. Compared with the experimental data, the relation between the relative density of EMWM and stress is approximately exponential, and then the relative density term *R*(*ρ_r_*) can be expressed as

(15) $$R(\rho_{r})=\exp\left[A(\frac{\rho_{r}}{\rho_{r0}}\;-\;1)\right]$$

where *ρ_r0_* is the reference relative density, *ρ_r0_* = 0.22. *A* is a fitting parameter. In this research, *A* = 2.46.

### 4.3. Effect of the Strain Rate

The stress–strain curves of EMWM under different impact velocities overlap highly. After comparison and analysis with experimental data, the relation between strain rate and stress is approximately linear, and then the strain rate enhancement term $M(\varepsilon ,\dot{\varepsilon })$ can be expressed as

(16) $$M(\varepsilon ,\dot{\varepsilon })=B\dot{\varepsilon }+C$$

where *B* and *C* are fitting parameters.

The strain rate enhancement term was fitted, and the strain rate enhancement term is given by

(17) $$M(\varepsilon ,\dot{\varepsilon })=0.00323\dot{\varepsilon }+0.9129\;\;\mathrm{for}\;{33.33\;s}^{-1}\leq \dot{\varepsilon }<{100.00\;s}^{-1}$$

and

(18) $$M(\varepsilon ,\dot{\varepsilon })=0.01617\dot{\varepsilon }\;-\;0.3643\;\;\mathrm{for}\;{100.00\;s}^{-1}\leq \dot{\varepsilon }\leq {133.33\;s}^{-1}$$

### 4.4. Constitutive Model Verification

The constitutive model for EMWM under low-velocity impact can be obtained by the combination of Equations (16)–(18). Therefore, the constitutive model for EMWM can be expressed as

(19) $$\sigma (\varepsilon )=R(\rho_{r})M(\varepsilon ,\dot{\varepsilon })f^{\prime }(\varepsilon )=\exp\left[A(\frac{\rho_{r}}{\rho_{r0}}-1)\right](B\dot{\varepsilon }+C)\frac{D_{0}\varepsilon^{2}}{{\left(1-\varepsilon \right)}^{2}}$$

where *ρ_r0_* is the reference relative density, *ρ_r0_* = 0.22.

The values of the other parameters are presented in Table 7.

A modified constitutive model for EMWM under low-velocity impact is established based on the Sherwood–Frost model. It contains the modified shape function *f*′(*ε*), the relative density term *R*(*ρ_r_*), and the strain rate enhancement term $M(\varepsilon ,\dot{\varepsilon })$.

**Figure 13:**
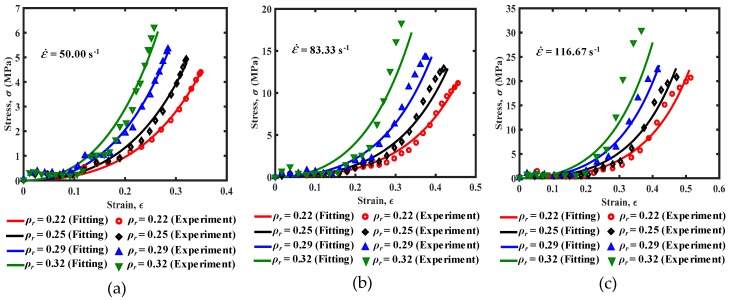
The comparison of stress–strain curves under different strain rates, (**a**) $\dot{\varepsilon }$ = 50 s^−1^, (**b**) $\dot{\varepsilon }$ = 83.33 s^−1^ and (**c**) $\dot{\varepsilon }$ = 116.67 s^−1^.

To evaluate the constitutive model, the calculation results are compared with the experimental results. The comparison results are shown in Figure 13. It can be seen from Figure 13 that the calculated stress–strain values of the EMWM with different relative densities matched well with the measured data. Although the deviation between the calculated data and measured data is larger for the EMWM with the relative density of 0.32, it can still predict its change trend. The comparison results show that the established model has high parameter identification accuracy and can well describe the mechanical properties of the EMWM under low-velocity impact.

## 5. Conclusions

In this paper, the low-velocity impact behaviors of EMWM with different relative densities were investigated through a series of drop-hammer impact tests. The effect of impact velocity and relative density of EMWM on mechanical properties and energy absorption of EMWM were studied. Moreover, a semi-empirical model was established to predict the dynamic compressive mechanical properties of EMWM under low-velocity impact loading. The main conclusions from this work are as follows:

(1) The impact energy absorption capacity of EMWM is strong, and the energy absorption rate is between 50% and 85%. The energy absorption capacity of EMWM decreases with the increase in density.
(2) The EMWM with the high relative density has excellent characteristics of repetitive energy absorption. Low-density EMWM will undergo plastic deformation under impact load.
(3) With the increase in relative density, the maximum deformation of EMWM decreases gradually, and the impact force of EMWM increases gradually. With the increase in impact-velocity, the phenomenon of stiffness softening before reaching the maximum deformation of EMWM becomes more and more obvious.
(4) The established constitutive model for EMWM under low-velocity impact can predict the dynamic compressive mechanical properties of EMWM under low-velocity impact loading.

Although the test results can qualitatively show that air damping and plastic deformation influence the energy dissipation characteristics of EMWM, the test method of this paper cannot provide quantitative analysis, which is also the focus of our work in the future.

## Figures and Tables

**Table 1:** The dimensions and structure parameters of EMWM specimens.

Batch	Specimen Name	Mass (g)	Height (mm)	Diameter (mm)	Relative Density	Batch	Specimen Name	Mass (g)	Height (mm)	Diameter (mm)	Relative Density
1	EMWM1_1	1190.21	61.42	120.91	0.218	3	EMWM3_1	1529.48	61.34	120.54	0.289
	EMWM1_2	1189.76	60.37	121.08	0.221		EMWM3_2	1525.70	59.26	120.48	0.280
	EMWM1_3	1189.54	60.83	121.02	0.220		EMWM3_3	1533.26	60.05	120.48	0.287
	EMWM1_4	1188.75	60.06	120.99	0.222		EMWM3_4	1532.18	60.16	120.51	0.286
	EMWM1_5	1188.27	61.63	121.10	0.217		EMWM3_5	1529.82	59.79	120.52	0.287
	CI	[1188.33, 1190.28]	[60.03, 62.69]	[120.93, 121.11]	[0.217, 0.222]		CI	[1526.46, 1533.72]	[59.17, 61.07]	[120.47, 120.54]	[0.282, 0.290]
2	EMWM2_1	1358.63	60.10	120.19	0.254	4	EMWM4_1	1702.34	61.62	120.11	0.310
	EMWM2_2	1362.46	59.83	120.18	0.256		EMWM4_2	1699.80	61.51	120.16	0.310
	EMWM2_3	1362.18	59.57	120.26	0.257		EMWM4_3	1701.48	62.27	120.09	0.307
	EMWM2_4	1361.44	59.81	120.18	0.256		EMWM4_4	1700.49	61.08	120.16	0.313
	EMWM2_5	1358.90	60.66	120.21	0.252		EMWM4_5	1703.59	60.67	120.17	0.315
	CI	[1358.45, 1362.99]	[59.47, 60.51]	[120.16, 120.24]	[0.253, 0.257]		CI	[1699.68, 1703.40]	[60.68, 62.17]	[120.09, 120.18]	[0.307, 0.315]

**Table 2:** The impact velocities corresponding to impact energies and strain rates.

**Impact velocity (m/s)**	2	3	4	5	6	7	8
**Impact energy (J)**	152	342	608	950	1368	1862	2432
**Initial strain rate (s^−1^)**	33.33	50.00	66.67	83.33	100.00	116.67	133.33

**Table 3:** Energy absorption rate for EMWM1 (batch1) under different impact velocities.

Specimen Code	Relative Density	Energy Absorption Rate						
		2 m/s	3 m/s	4 m/s	5 m/s	6 m/s	7 m/s	8 m/s
EMWM1_1	0.218	0.744	0.744	0.687	0.743	0.741	0.817	0.849
EMWM1_2	0.221	0.762	0.757	0.685	0.747	0.741	0.821	0.835
EMWM1_3	0.220	0.760	0.734	0.695	0.744	0.729	0.806	0.849
EMWM1_4	0.222	0.758	0.735	0.702	0.740	0.748	0.811	0.852
EMWM1_5	0.217	0.744	0.711	0.677	0.742	0.753	0.817	0.819
MV	0.220	0.754	0.736	0.689	0.743	0.742	0.814	0.841
STD	0.002	0.009	0.017	0.010	0.003	0.009	0.006	0.014
CI	[0.217, 0.222]	[0.743, 0.764]	[0.715, 0.757]	[0.677, 0.701]	[0.739, 0.746]	[0.731, 0.753]	[0.807, 0.821]	[0.823, 0.858]

**Table 4:** Energy absorption rate for EMWM2 (batch2) under different impact velocities.

Specimen Code	Relative Density	Energy Absorption Rate						
		2 m/s	3 m/s	4 m/s	2 m/s	6 m/s	7 m/s	2 m/s
EMWM2_1	0.254	0.734	0.700	0.689	0.715	0.725	0.765	0.830
EMWM2_2	0.256	0.731	0.704	0.680	0.720	0.749	0.766	0.841
EMWM2_3	0.257	0.753	0.707	0.679	0.730	0.723	0.773	0.848
EMWM2_4	0.256	0.740	0.714	0.641	0.720	0.727	0.765	0.849
EMWM2_5	0.252	0.743	0.698	0.645	0.710	0.728	0.798	0.847
MV	0.255	0.740	0.705	0.667	0.719	0.730	0.773	0.843
STD	0.002	0.009	0.006	0.022	0.007	0.011	0.014	0.008
CI	[0.253, 0.257]	[0.730, 0.751]	[0.697, 0.712]	[0.639, 0.694]	[0.710, 0.728]	[0.717, 0.744]	[0.756, 0.791]	[0.833, 0.853]

**Table 5:** Energy absorption rate for EMWM3 (batch3) under different impact velocities.

Specimen Code	Relative Density	Energy Absorption Rate						
		2 m/s	3 m/s	4 m/s	2 m/s	6 m/s	7 m/s	2 m/s
EMWM3_1	0.280	0.722	0.677	0.665	0.732	0.717	0.736	0.806
EMWM3_2	0.289	0.740	0.672	0.677	0.737	0.706	0.748	0.806
EMWM3_3	0.287	0.741	0.691	0.631	0.739	0.714	0.736	0.821
EMWM3_4	0.286	0.709	0.675	0.653	0.755	0.741	0.756	0.810
EMWM3_5	0.287	0.719	0.670	0.678	0.745	0.731	0.781	0.850
MV	0.286	0.726	0.677	0.661	0.742	0.722	0.751	0.819
STD	0.003	0.014	0.008	0.020	0.009	0.014	0.019	0.019
CI	[0.282, 0.290]	[0.709, 0.743]	[0.667, 0.687]	[0.637, 0.685]	[0.731, 0.753]	[0.704, 0.739]	[0.728, 0.774	[0.796, 0.842]

**Table 6:** Energy absorption rate for EMWM4 (batch4) under different impact velocities.

Specimen Code	Relative Density	Energy Absorption Rate						
		2 m/s	3 m/s	4 m/s	2 m/s	6 m/s	7 m/s	2 m/s
EMWM4_1	0.310	0.696	0.644	0.592	0.514	0.680	0.726	0.823
EMWM4_2	0.310	0.702	0.643	0.591	0.547	0.673	0.713	0.814
EMWM4_3	0.307	0.700	0.656	0.627	0.538	0.674	0.716	0.812
EMWM4_4	0.313	0.711	0.651	0.623	0.575	0.726	0.730	0.810
EMWM4_5	0.315	0.701	0.667	0.622	0.552	0.682	0.712	0.814
MV	0.311	0.702	0.652	0.611	0.545	0.687	0.719	0.815
STD	0.003	0.006	0.010	0.018	0.022	0.022	0.008	0.005
CI	[0.307, 0.315]	[0.695, 0.709]	[0.640, 0.664]	[0.589, 0.633]	[0.518, 0.573]	[0.660, 0.714]	[0.709, 0.729]	[0.808, 0.821]

**Table 7:** Parameters of the constitutive equation.

Parameter	*A*	*D*_0_	${33.33\;s}^{-1}\leq \dot{\varepsilon }<{100.00\;s}^{-1}$		${100.00\;s}^{-1}\leq \dot{\varepsilon }\leq {133.33\;s}^{-1}$	
			*B*_1_	*C*_1_	*B*_2_	*C*_2_
Value	2.46	13.95 MPa	0.0023	0.9129	0.01617	−0.3643
